# Between Social Inclusion and Exclusion: Integration of Daycare Guests in the Nursing Home Setting

**DOI:** 10.1093/geront/gnaa157

**Published:** 2020-10-09

**Authors:** Kerstin Hämel, Gundula Röhnsch

**Affiliations:** Department of Health Services Research and Nursing Science, School of Public Health, Bielefeld University, Bielefeld, Germany

**Keywords:** Integrated care, Long-term care, Nursing home residents, Qualitative study, Social participation

## Abstract

**Background and Objectives:**

In integrated daycare, community-dwelling older people in need of care join existing groups in residential care facilities during the day. This study focuses on how nursing home residents experience the integrative care approach, exploring opportunities for social inclusion and mechanisms of exclusion.

**Research Design and Methods:**

A purposive sample of residents differing in cognitive capacity and level of (non)conflictual interaction with daycare guests was selected. Episodic interviews with residents (*N* = 10) and close relatives (*N* = 2) were conducted in 3 pilot facilities in Germany and analyzed using thematic coding.

**Results:**

The analysis revealed different orientation patterns towards the presence of daycare guests: respondents (a) demonstrated indifference to the daycare guests, (b) saw bonding with guests as a means to connect to the outside world, and (c) perceived incompatibility between in-group and out-group. Criticisms included disruption of daily routines and loss of privacy. Most interviewees came to terms with the care situation using rational and moral arguments.

**Discussion and Implications:**

The study reveals the importance of residents’ participation when integrating daycare guests. Institutional procedures are required to prevent exclusion of daycare guests and avoid overtaxing residents.

The potential of integrated care models has been discussed intensively in health policy and health services development for many years (World Health Organization [WHO], 2015, 2016). This care model is seen as providing more people-centered care by supporting continuity and improving access, quality, and user satisfaction (Baxter et al., 2018). Integrated care models can enable more person-centered care by bridging rigid institutional demarcations (e.g., providing health and social services) (Hämel & Röhnsch, 2019; van Wijngaarden et al., 2006); at the same time, they promote a holistic perspective and expand the options available to care professionals for addressing users’ needs (Schaeffer & Hämel, 2016). Abbott and colleagues (2018) also stress that a “person-centered philosophy of care” is characterized by care professionals understanding their clients’ social “preferences, goals and values” (p. 1126) and making these the basis for shaping care processes (Abbott et al., 2018; cf. Van Haitsma et al., 2020). However, studies that elicit the users’ perspective on the existing integrated models are lacking (Baxter et al., 2018). This article focuses on the experiences of nursing home residents who have been affected by implementation of an integrated daycare model in their living environment.

Integrated daycare is a model for organizational integration (Kodner, 2009; Valentijn et al., 2013) of fully residential and daycare services for older people with long-term needs. It is based on an *integrative* care approach: Community-dwelling older people in need of care visit existing housing groups in residential care facilities close to their homes during the day. Nursing home residents and day guests spend the day together and jointly use services offered, for example, nursing and assistance, health counseling, catering, and social events (for further details, see “Research Design and Methods”). In Germany, integrated daycare services have been implemented by practice-driven concept development: They have been implemented in individual nursing homes on the basis of needs identified in practice, but without significant academic or political attention or support; data on the prevalence of integrated daycare in Germany are not available. Our own research suggests that integrated daycare is applied in isolated cases primarily in southern Germany (Cramer & Schönberg, 2019; Hämel & Röhnsch, 2019). The rationale behind integrated daycare is to improve access to daycare services for older people by opening-up nursing homes. The model also endeavors to offer a seamless transition to inpatient care if this becomes necessary (Hämel & Röhnsch, 2019). Although integrated daycare may deeply affect the lifeworld(s) of residents, their perception of it has yet to be studied.

Research based on a survey of key informants and professionals with experience in implementation integrated daycare determined that it offers specific benefits for care users, especially when implemented as a community-oriented model based on an integrative care approach: Using shared services makes it easier for both home residents and day visitors to cultivate new social contacts in their neighborhood and community (Hämel & Röhnsch, 2019; Röhnsch & Hämel, 2019). This is particularly interesting, as nursing homes have long been denounced as “closed institutions” that neglect the social and cultural needs of residents (Hämel, 2012). That criticism echoes Goffman’s concept of the “total institution” with its heavy restrictions on residents’ individual expression (Goffman, 1961). Residents of care homes have always been vulnerable to exclusion from equal participation, from the general standard of living, and from society’s ideal of a good life (Hämel, 2012). In Germany, civil society initiatives are working to overcome the culture of isolation in nursing homes by opening them up and integrating them into the local community. Here, opening homes to the outside world can be seen as a step towards opening them internally—to their residents (Hämel, 2012, 2016). Several studies indicate that nursing homes are still strongly inclined to prioritize institutional requirements and routines over residents’ needs (Amrhein, 2005; Hämel, 2016; Koch-Straube, 2005; Paddock et al., 2019). As such, they represent “instances of exclusion of modernity” (Stichweh, 2010), separating people with disabilities from the rest of society. However, the academic discourse tends to overlook that nursing homes *also* provide inclusionary spaces that facilitate participation for people with special needs. This is even more true today, as nursing home care concepts propagate person-centered care in a home-like living environment that balances privacy and opportunities for social interaction, and provides its users with the means to exert influence and participate (cf. Dee & Hanson, 2019).

In order to analyze perceived benefits and challenges of integrated care, a specific characteristic of long-term care institutions must be taken into account: From the perspective of residents, the nursing home is not simply a facility that provides a range of services; it is the environment that shapes their daily life, including social relations (Rijnaard et al., 2016). Studies in nursing homes have shown repeatedly that relationships between residents are essential for well-being and perceived quality of life (Kang et al., 2019; Roberts & Bowers, 2015). Research also shows how difficult social contacts in homes can be, and how widespread experiences of loneliness are (Paque et al., 2018). Residents rarely succeed in establishing meaningful personal relationships with each other (Hauge & Kristin, 2008; Knight & Mellor, 2007). The community formed within a nursing home is considered “forced” (Hauge & Kristin, 2008). Where many residents have little in common except diseases and disabilities, communication is superficial and limited to health issues (Bonifas et al., 2014; Hauge & Kristin, 2008).

Integration of daycare guests may provide home residents with opportunities to foster (new) social contacts and interact with the outside world. The presence of daycare guests in the institutional care setting also creates challenges, however. Caregivers have to address the potentially different needs of residents and daycare guests. In addition, nursing home residents describe increasing turnover due to short-term rehabilitation stays as a barrier to meaningful relationships (Kang et al., 2019). Finally, it becomes particularly difficult for professionals in the nursing home setting to support peer relationships when users suffer from psychiatric conditions such as dementia or anxiety disorders. Such conditions impede the functioning of groups and are associated with a high level of care (Hämel & Röhnsch, 2019).

## Aim of the Study

This study focuses on how nursing home residents experience the presence of daycare guests in “their” facilities. We examine residents’ attitudes and (social) orientation towards daycare guests to construct an initial knowledge base about (non)acceptance of the integrated daycare model from the perspectives of home residents. Of particular interest is:

1. To what extent do residents integrate daycare guests in their daily routines?
2. How do residents discuss social contact and separation in this context?
3. To what extent does the presence of daycare guests affect how residents participate and exert influence?

We pay particular attention to opportunities for social inclusion and mechanisms of exclusion. Identifying residents’ perceptions and experiences is central to an adequate assessment of integrated daycare.

## Research Design and Methods

This qualitative study is part of an evaluation of the model project “Rethinking long-term care institutions.” In this project, nursing homes in the German state of North Rhine–Westphalia endeavored to expand to become integrated local care centers. Three of these centers added integrated daycare to their services.

### Research Setting: Nursing Homes and Integrated Daycare

In terms of resident profile and managing organization, the three nursing homes are relatively similar and also typical for Germany (see Table 1). In terms of nursing homes for older people in Germany as a whole, about 69% of residents are affected by some form of dementia (Schäufele et al., 2013).

**Table 1.** Provider Type and Population Characteristics of Nursing Homes in Germany and of the Three Investigated Homes^a^

	Nursing home 1	Nursing home 2	Nursing home 3	Germany, total^b^
Provider type	Nonprofit	Nonprofit	Nonprofit	Nonprofit: 54% Forprofit: 41% Public: 5%^c^
Number of residents	78	87	84	Total: 818,289
				Mean: 71
Gender of residents				
Male	25.6%	17.2%	21.4%	29.6%
Female	73.1%	82.8%	78.6%	70.4%
Age groups^d^				
Younger than 75	2.6%	5.7%	4.8%	17.3%
75–79	12.8%	3.4%	15.5%	12.8%
80–84	21.8%^e^	18.4%	25.0%	19.7%
85–89	20.5%^e^	20.7%	20.2%	23.8%
90 and older	42.3%	51.7%	34.5%	26.4%
Care levels of residents^f^				
Care level 1	–	–	–	0.9%
Care level 2	12.8%	14.9%	11.9%	21.3%
Care level 3	37.2%	35.6%	29.8%	31.5%
Care level 4	33.3%	31.0%	42.9%	29.4%
Care level 5	15.4%	18.4%	15.5%	16.2%
Care level to be determined	–	–	–	0.7%

*Notes*: Authors’ calculation using data from pilot facilities (nursing homes 1–3) and official care statistics (amtliche Pflegestatistik; Germany, total; Statistisches Bundesamt, 2018).

^a^As of pilot facilities December 31, 2018; Germany overall, December 15, 2017. ^b^Including respite care. ^c^Provider figures for long-term fully residential care. ^d^Differences in age structure between total nursing home population and pilot facilities reflects specialization of pilot facilities in care of older people. ^e^For nursing home 1, only an aggregate figure for residents aged 84/85 was available. The number of residents aged 84/85 was distributed equally to the categories 80–84 and 85–89 years. ^f^In Germany, five care levels are defined to determine eligibility for long-term care and the level of benefits paid by the Long-Term Care Insurance. The higher the care level, the greater the need for personal assistance and nursing care increases (cf. Büscher et al., 2011; Nadash et al., 2018).

Like all nursing homes in Germany, the nursing homes in our study offer room, full board and (nursing) care services. The care services encompass basic care (assistance with personal hygiene, dressing/undressing, toilet, activities of daily living, instrumental activities of daily living) and technical nursing care (wound management, medication). Nursing homes offer residents’ individual support (advice and discussions), group activities (music-making, memory training, group cooking), and organized social/cultural events (seasonal festivals). Nursing homes’ service provision and their remuneration are negotiated bilaterally between the associations of the sickness funds (long-term care insurers) and the provider associations (Nadash et al., 2018); while the system guarantees fairly standard quality of care in nursing homes in Germany, the quality of board and accommodation varies more widely, as does the breadth of sociocultural activities. A person in need of care may choose any nursing home operating within the Long-term Care Insurance Act (Nadash et al., 2018). In Germany, the Long-term Care Insurance Act guarantees (nearly) universal coverage (by compulsory insurance); 89% of the German population are insured by statutory care insurance, and 11% by private insurance (Nadash et al., 2018; cf. Greß et al., 2020). Each of the three studied nursing homes has six daycare places. The staffing ratio was increased using a notional figure of one full-time post per five daycare places; the increase was distributed in various proportions among the various types of staff (Hämel & Röhnsch, 2019). Daycare guests are entitled to use all the home’s services and facilities (although use of bodily care such as bathing or wound management was the exception).

Integrated daycare permits greater flexibility in scheduling than the daycare-only facilities that are more common in Germany (and the United States). Guests can come and go relatively flexibly, as their presence is less contingent on specific opening hours (Cramer & Schönberg, 2020; Hämel & Röhnsch, 2019). However, late evening and weekend attendance was possible only occasionally and by arrangement (Hämel & Röhnsch, 2019).

### Data Collection: Episodic Interviews

Data were collected using episodic (semistructured) interviews (Flick, 2018). In contrast to narrative interviews, episodic interviews focus on certain defined aspects; they seek to elicit episodes associated with specific phenomena rather than comprehensive life stories (cf. Flick, 2018; Mueller, 2019). The questions asked during the interview process restore focus help to avoid overtaxing respondents with limited communication skills. Episodic interviews thus also appear suitable for people with dementia. This study respects the autonomy of persons with dementia by including their personal experiences and perspectives—an important ethical aspect of evaluation (Baalen et al., 2010, p. 120).

### Interview Guideline

The interview guideline encompassed: daily procedures in inpatient care; personal experience of integrated daycare; social relationships with daycare guests and other residents, perceived changes in procedures due to the presence of daycare guests, and experience of nursing care.

The following example illustrates the combination of targeted questions and narrative prompts that is typical for the episodic interview (area “personal experience of integrated daycare”):

Interviewer: “So do you think you can influence how you spend the day here in the home and what you do with the other residents and the guests? Can you tell me about a situation that illustrates this?”

In cases where family members were interviewed due to residents’ cognitive impairments and/or spatial and temporal disorientation, the guideline was adapted accordingly. Relatives were asked to describe the resident’s perceptions as they understood them. They were also asked for their own assessment of integrated daycare. Apart from minor reformulations for relatives, all participants were asked the same questions (see guidelines for interviewing residents and relatives of home residents provided as Supplementary Material).

### Access and Sampling

Nursing home residents were approached by care facility staff according to the sampling criteria (see below). Prior to the interviews, participants were provided with verbal and written information about the study and its data protection measures. All participants signed an informed consent statement.

**Figure 1.**
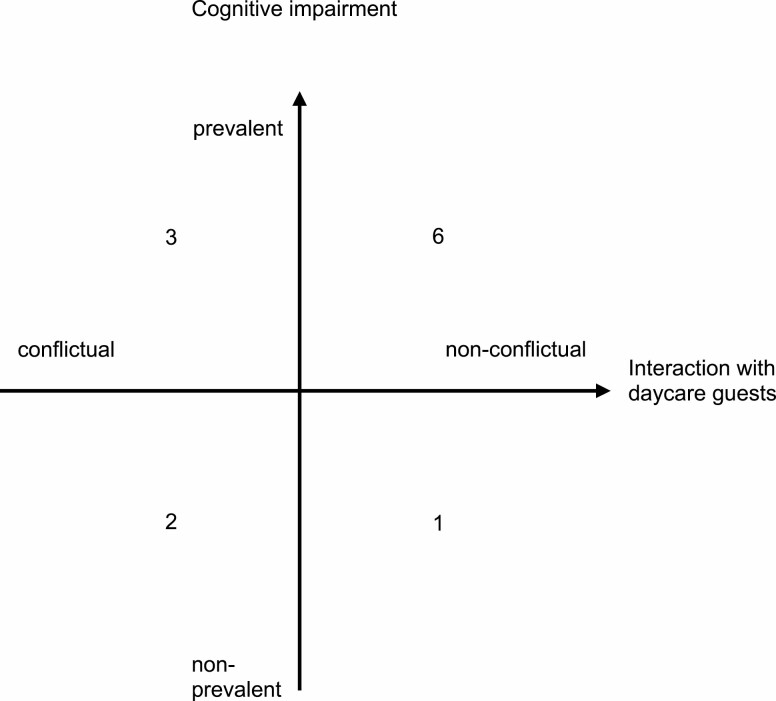
Characteristics of the study participants (sampling criteria).

In order to take into account variation in (personal) background and context, the sample was compiled for maximum contrast. The goal was to select residents who—according to pre-assessment by nursing home staff—differed in cognitive capacity (impairment) and in intensity of (non)conflictual interaction with daycare guests. The latter criterion was also chosen to increase the diversity of the sample. Figure 1 shows the structure of the sample.

Residents who were no longer able to express themselves verbally and/or suffered spatial and attention disorientation were excluded. In these cases, family members were asked to participate on behalf of the resident. Twelve interviews were conducted: 10 with residents and 2 with family members (see Table 2). Four residents participated in each of the three pilot facilities. The interviews were conducted in November 2018.

**Table 2.** Study Participants: Age and Gender

	Gender		
Age (in years)^a^	Male	Female	Total
75–79	1	1	2
80–84	1	1	2
85–89	–	2	2
90–94	1	4	5
95–99	–	1	1
*N*	3	9	12

*Note*: ^a^In cases where a family member was interviewed on behalf of a resident, the information refers to the resident.

### Data Analysis: Thematic Coding

The interviews lasted 20–50 min and were transcribed in full and anonymized. The interviews were evaluated using MAXQDA 12 based on thematic coding developed by Flick (2018). Thematic coding integrates principles from case analysis with cross-case comparisons to create an overarching thematic structure.

In the first step, we developed a category system for individual cases (interviews), using the interview guideline as the basis for deductive creation of general categories. Interview material was assigned to descriptive keyword-style categories and openly coded (Strauss’s coding paradigm) by breaking up the text using sensitizing questions (How ...? Why ...? etc.) (Strauss, 1987; cf. Flick, 2018). This allowed us to inductively code (e.g., introducing the category “accepting the presence of daycare guests”) and generate a thematic structure for the individual cases (cf. Flick, 2018). Relationships between individual codes were established under the coding paradigm (cf. Strauss, 1987).

In the second step, we looked for similarities and differences between case-specific structures and examined their relationships. In the process, individual categories were fine-tuned, dimensionalized and raised to a higher level of abstraction (e.g., merging the categories “acceptance of presence of daycare guests” and “negative aspects of presence of daycare guests” into the category “moral and rational considerations”).

The outcome was an overarching thematic structure comprising five categories (see Table 3). Finally, each category was subjected to fine analysis with detailed interpretation of individual text passages (e.g., narrations of situations concerning the extent to which the presence of daycare guests disrupts the usual routine) (cf. Flick, 2018).

**Table 3.** Attitudes of Nursing Home Residents Towards the Presence of Daycare Guests: Definitions and Examples

Category (orientation towards daycare guests)	Definition	Example
Expressing indifference (neutral)	Interviewee does not mind who they spend their time with, does not care who their peers are or where they come from. Formal criteria such as group membership are not relevant for personal affinity.	“If they [daycare guest] speak to a [resident] and the resident doesn’t want that then you withdraw (…) and with the next [daycare guest], then you think you know them already I don’t know how long.” (Ms. Dehmel)
		“Well they get taken away every evening or collected by their relatives. People see it differently. For me it’s normal. They go home now, that’s how it is.” (Ms. Oswald)
Being connected to the outside world (rather positive)	Interviewee perceives daycare guests as a link to the outside world. Presence of daycare guests contributes to variety and counteracts monotony. Guests also bring news from the neighborhood and wider world.	“Why should I mind?! One of them comes from the village, she’s blind, I’ve known her since childhood. I know several of them, the ones who come. It’s all fine. I don’t know why anyone should mind them coming.” (Ms. Leistner)
		“(…) well maybe its sometimes enriching to step out of the routine (…) then it’s ‘Oh look, thank God someone’s coming, someone to communicate with’ (…)” (Mr. Prim)
In-group and out-group (rather negative)	Interviewee emphasizes differences and incompatibility of behavior, interests, needs and problems between daycare guests and nursing home residents. Interviewee feels sense of belonging as a nursing home resident and distances from daycare guests as a group.	“And there’s one sitting there, head back, mouth wide open. I said it before, let’s get a little bird and put it in there. There’s a few of them [daycare guests]. One just sits there, gob wide open. Terrible it is.” (Ms. Kirsch)
		“Well, some [daycare guests] are a bit particular in that way and some of them can’t cope at all [here] and some others they are completely deaf (laughs). Sometimes they’re quite sick and that’s sometimes not very nice.” (Ms. Kallms)
Sensing disruptions (negative)	Interviewee perceives daily routines interrupted or changed due to presence of daycare guests. Perceives daycare guests as “intruders” who threaten familiar atmosphere and personal space.	“Well only that the day guests started coming and (…) it was a bit louder than normal. Before we used to all sit here together and had coffee and ate breakfast, lunch, and that tends to get a bit forgotten now.” (Mr. Prim)
		“Well if you’re asking about things that have changed recently, sometimes you get annoyed when visitors (…) just open the door and come in. They say it’s their room here and they can be here and go to the toilet here (…) I can only speak for myself. I don’t like that at all. I can’t stand that.” (Ms. Harnack)
Rational and moral considerations (neutral/negative)	Interviewee accepts the presence of daycare guests on the basis of rational and moral arguments—but in certain situations only reluctantly. Acceptance is based on subjective understanding of the situation of the daycare guests and the wish to avoid (open) conflicts in the nursing home.	“There’s no point in me seeing it negatively and getting worked up. that’s just how it is. They aren’t here because they want to be. If it doesn’t work out with the housework or because of the children’s jobs.” (Mr. Liebel)
		“It doesn’t interest me really. We have our territory. We sleep here, we get fed here, we live here and they just come for the day and get picked up in the evening.” (Ms. Tewes)

All interview data were coded in the manner described above. Coding was conducted by the second author in very close and frequent consultation with the first author. Ambiguities and inconsistencies in coding interview material and assigning codes to categories were discussed until a consensus was reached; where necessary the coding was revised.

### Ethics

The Ethics Committee of Bielefeld University approved the study (file no. EUB 2018-152). The assessment was carried out according to the ethics guidelines of the Deutsche Gesellschaft für Psychologie e.V. All interviewees gave written consent for the use of their interviews.

## Results

All interviewed residents felt content and secure in their care facility and enjoyed living there. Their well-being is attributable largely to the professional care and the wide range of activities offered during the day. Some interviewees, however, mentioned that the carers generally had little time (for them). The interviewees’ generally positive attitude towards their nursing home was not negated by the presence of daycare guests.

We were able to identify five categories of attitude and orientation towards the presence of daycare guests. The five patterns represent (rather) positive, negative, or neutral attitudes towards daycare guests; they are nonexclusive. Table 3 gives an overview. In the following, we describe them in greater detail.

### Expressing Indifference

Residents’ attitudes concerning the daycare guests’ presence are influenced by general feelings of personal sympathy and/or antipathy. It appears to be crucial whether they bond with the day visitors, for example whether they find topics of mutual interest. It is not unusual for interviewees to develop a closer relationship to some guests than others: “(...) there’s always someone you don’t like so much, they can be avoided” (Ms. Dehmel).

Many respondents seem indifferent to the presence of daycare guests. They have become accustomed to it. The interviewees appear to not even notice them as a specific group in the nursing home. It is simply “alright” when the guests are there. Some interviewees appear largely unaware who is a resident and who a day guest—they expect frequent change in caregivers and the people they see daily.

So, I do not know who is new. One knows everybody. One talks to everyone. There are so many altogether, and it’s different every time. (Ms. Tewes)

Although respondents often indicated indifference to the presence of daycare guests, they also clearly identified positive and/or negative aspects of socializing with the guests. In the following, the arguments underlying the different assessments are discussed in more detail.

### Being Connected to the Outside World

Six of the 12 interviewees mentioned positive aspects of the presence of the daycare guests and appeared motivated by a desire to connect to people.

Those who had until recently been living alone and had been unhappy with the situation enjoyed socializing: “(...) the more people there are, that’s always good. I like being with people” (Ms. Leistner). It appears secondary to them whether the people they socialize with are residents or daycare guests; what matters is being socially involved.

Those who had lived longer at the care facility seem to view the company of daycare guests as a welcome change from a possibly monotonous everyday routine. The guests are seen as a link to the outside world, as an opportunity, “(…) perhaps [to] have other conversations” (Ms. Buchholz). The interviewed relatives made similar observations. The day guests introduce new topics of conversation that would not otherwise arise. They enable the residents to participate in the proximate social environment as well as in world events per se, thus giving them a sense of normality.

It’s an asset. You hear more about the outside then (...) that’s how it is, we don’t pick up a lot about what’s going on in the world. (Mr. Prim)

The interviewees sense bonds with daycare guests, especially if they used to know (of) each other and have shared experiences to recall—joint activities, old friendships, or places they once lived. In this way, daycare visitors can help residents maintain a sense of biographical continuity.

One variant of positive attitude towards daycare visitors may also result in critical perspectives on their own situation as a resident. Some respondents remarked on virulent feelings of boredom and monotony *because* the day guests are privileged to return to their familiar surroundings, while they themselves must stay in the facility. Envy and resentment can trigger both appreciation of being able to stay at the facility in the evening and a desire to return home: “(...) that means then, ‘Why don’t you have to go home, when I have to’. Or: ‘Look, I can go home and you have to stay here’” (Ms. Harnack).

### In-group and Out-group

Eight interviewees *also* regard the day visitors from a critical perspective. They seem to believe that the interests, needs and problems of the in-group (residents) and the out-group (daycare guests) are, in part, incompatible. Daycare guests are seen as strangers, as “intruders” with their own interests. In part, it also appears to the respondents to be completely inexplicable why the day visitors are residing in “their” facility.

Is it necessary? I wonder what the [day visitors] want? I keep myself occupied. There’s nothing one can say, they are different (...) how did they get to come here, and how were they chosen? That’s what kind of worries one a bit. (Mr. Felmy)

The described “otherness” of daycare guests also manifests itself in their apparent lack of interest in belonging to the residents’ community. They block any closer contact with residents and take part in joint activities sporadically rather than regularly. According to the interviewees, the day guests have only themselves to blame if they remain the outsiders “(...) we have tried to integrate them [the day guests], but if they don’t want to, then we can’t do anything about it” (Mr. Liebel).

### Sensing Disruptions

Interviewees who expressed negativity regarding the day guests linked their presence with “disruption” of everyday life and the (threatened) loss of shared familiarity and privacy. These residents mentioned higher noise levels caused by daycare visitors, which make it difficult for them to converse in a normal voice. They complained that it spoils the usually cozy atmosphere, for example at meals. This was even more the case when day visitors are agitated and residents are confronted with inappropriate or irrational behavior.

Interviewed residents attributed the disruption associated with daycare guests as a result not only of guests’ behavior, but also of facility procedures that they associate with the guests’ presence. Specifically, the evening collection of guests to take them “home” was said to disrupt residents’ daily routines and leave them with nothing to do.

Sometimes they [daycare guests] join us when we have the [social activities]. But then they (...) have to leave in the middle (...), the first get up and leave again. Then the activity is interrupted, then we also take a break. (Ms. Kirsch)

Some interviewees are unsettled by guests’ personal idiosyncrasies. Interestingly, these respondents feel uncomfortable about behaviors and habits that are not specific to the guests, but could just as well be found among residents. But they do not reflect on this: “(...) we want lunch, and [particular day guest] puts his dentures on the table” (Ms. Oswald).

Another respondent complained that daycare guests suddenly appeared in her room as if it was their own. She considered such an “intrusion” into her personal space very unpleasant. It made her feel helpless. “(...) they think that when we withdraw to a room, then they can do that, too. They think it belongs to the general public” (Ms. Harnack). She blamed these visits to her room on the lack of a specified room to which day guests retreat. Moreover, she felt obliged to converse with them, as if she were their host. As a result, she felt pushed into the role of a social worker by the daycare guests, which she found challenging, especially when confronted with certain “disease-specific” behavior (e.g., frequent repetition of the same questions). She also found it overwhelming when daycare guests responded to her efforts to integrate them into everyday life at the facility with disinterest, while, at the same time, demanding more attention from her.

(...) I spent a lot of time with the day guests. Until I realized that they don’t want anything explained to them. And then I thought to myself, they are not my responsibility (...) only then, they follow you, and run after you wherever you go. (Ms. Harnack)

Ms. Harnack also said that the quality of residents’ nursing care had deteriorated due to the presence of the daycare guests. She believed that nursing staff were no longer able to offer the level of care the residents needed, for example that they had insufficient time for those unable to attend to their personal hygiene because they also had to take care of the day guests.

### Rational and Moral Considerations

Those who regard the presence of day visitors at least partly critically—albeit cautiously and indirectly—seem to have found a way to accept the situation. In order to deal with the uneasiness occasionally induced by the presence of daycare visitors, they seek rational explanations and treat the visitors’ presence as inevitable. For example, they try to understand the day guests’ family situation better and develop a sympathetic attitude toward family members seeking to ease their care burden. From the interviewees’ point of view, there is no alternative but to come to terms with the daycare guests. Despite such verbal efforts to “make their peace” with the presence of the day guests, the residents remain (surreptitiously) skeptical.

Denying concern appears to be, in part, a moral/normative phenomenon. Residents assume that the guests’ health situation is similar to their own, and hence, they should not criticize their presence. They seem to accept the day guests’ presence on the basis that they also need assistance: “(...) the people here are all ailing in some way” (Mr. Prim). Interviewees also appear to shy away from commenting critically on the presence of daycare guests because they assume getting on well with everyone at the care facility is in their own best interest in the long run. Quarrels and open conflicts would disrupt a harmonious and peaceful daily routine. So they put their own sensitivities to one side.

(...) sometimes there is something that you don’t like so much, but you always have to stay in the group. You cannot make a big thing of it, somehow. (Ms. Kallms)

## Discussion and Conclusions

In this interview study, we explored how nursing home residents experience the presence of daycare guests in their living environment. By analyzing the residents’ perspectives, our study provides a unique basis for understanding the dynamics of social affiliation and segregation in nursing home-based, integrated daycare. An understanding of these dynamics can be useful for configuring integrated daycare in a way that enables residents to realize their preferences in relation to the social context; this is a pivotal aspect to promote person-centered care (cf. Abbott et al., 2018). The analysis revealed various, sometimes contradictory attitudes and orientations towards the presence of daycare guests. In the following, we will discuss how nursing home residents exert influence and participation in this mixed-care model. Approaches to facilitate inclusion and counteract exclusion mechanisms in integrated daycare will be discussed.

1. Regular visits by daycare guests enable those residents who are generally interested in social bonding *to develop new social contacts and meaningful relationships*. In this way, daycare guests can counteract feelings of loneliness in the absence of meaningful relationships (Cho et al., 2017). Moreover, by building friendships with the guests, residents gradually integrate the day visitors into everyday life in the facility. In this way, residents find themselves able to influence social togetherness in their living environment and contribute to a positive atmosphere in the nursing home community (see also Roberts & Bowers, 2015).
2. Some respondents emphasized intractable differences between the groups of residents and guests. These residents seem to believe implicitly that the interests and needs of the day visitors differ from their own. Furthermore, if they sense that daycare guests are not interested in being integrated in the home community, these visitors are perceived as “intruders.” This suggests that the presence of daycare guests can unintentionally contribute to a *strengthening of the sense of togetherness* among (selected) home residents, who seem to agree that they need to stick together against the outsiders. These residents implied that they saw themselves as “the heart” of this care environment. This implied cohesion of the in-group appears to make it easier for the respondents to articulate their own interests in the design of care and to seek (and find) allies for this purpose, and thus expands their potential to (re-)gain control over their own situation. However, this orientation pattern also fosters division between the two user groups, which could lead to a gradual exclusion of daycare guests.
  In order to counteract such potential developments, a closer understanding of what concerns residents about daycare guests’ presence is essential: The interviewed residents discussed *disruptions of daily routines* caused by daycare guests. Certain respondents voiced feelings of helplessness caused by day visitors “turning up” unexpectedly in their private rooms, apparently not accepting or understanding their need for privacy. Others complained that structured social activities in the care facility were disrupted when visitors are picked up to be taken home. Both complaints imply a loss of self-determination: on the one hand, through lack of privacy, on the other, due to organizational necessities. Lack of privacy and loss of self-determination are recurring themes for nursing home residents and negatively affect their well-being (cf. Kloos et al., 2019; Knight & Mellor, 2007; cf. Abbott et al., 2018). Loss of autonomy also can exacerbate feelings of loneliness in care institutions (cf. Falk et al., 2013).
  Our findings indicate that the positive effects of the integrated daycare model are counteracted by factors that negatively affect the well-being of residents. This calls for a rethinking of the current institutional design of the integrated daycare model. In order to safeguard the residents’ need for privacy, private and public rooms need to be clearly identified. Daycare guests need to be provided with adequate spaces to retreat to during the day, and the residents’ private rooms designated off limits. Residents—as experts concerning their own living situation—also need to be actively involved in the development of institutional procedures for better integration of daycare guests, as they are directly affected by their presence. Such participation gives residents an opportunity to exercise autonomy, gain control over their lives and shape their living environment. This implies also that nursing home staff should be allowed to treat them as partners in decision-making concerning institutional procedures (Kang et al., 2019).

3. Interestingly, the interviewees often expressed *indifference to the presence of daycare guests*. Asked directly, they usually dismissed the idea that the presence of the daycare guests disturbed them in any way. Critical perceptions were chiefly derived from stories they told about their daily life and experiences. This implied compliance, politeness, and indifference toward social interaction corresponds to the findings of Knight and colleagues (2007). In our study, however, the respondents disclosed certain rational and moral arguments behind their apparent indifference. Our findings suggest that the respondents can identify with the guests and their need to be supervised and cared for in a nursing home setting. Residents suggested that it would be wrong to scorn or openly criticize the guests’ presence. This habit of avoiding confrontation seems to be a common mode of managing relationships for the respondents. Similar to reactions found in the study by Palacios-Ceña and colleagues (2014), nursing home residents appear to recognize the importance of not being labeled “difficult” for relationship building to ensure beneficial treatment (Palacios-Ceña et al., 2014). It can also be assumed that home residents who express indifference to the presence of daycare guests are thus (partially) renouncing any aspirations to influence their living environment actively. As Kardorff and Meschnig (2009) noted, the respondents belong to a generation for whom downplaying their own demands in care settings is understood as an expression of gratitude. On the one hand, this attitude opens opportunities for inclusion of daycare guests; on the other hand, nursing home staff need to ensure that residents’ needs are still met.

Overall, our analysis reveals that residents are interested in integrating daycare guests into their community, as long as they can co-determine joint activities and space usage. However, it is vital that they also have the freedom to retreat to a secure private space when needed. Our study indicates that nursing home residents also benefit from social interaction with daycare guests. So a well-considered opening-up of nursing homes in an integrated daycare model offers opportunities to ease the much criticized “closedness” of nursing homes, and may help to counteract the harmful effects of isolation and exclusion of home residents from local communities (Hämel, 2016).

Some residents try to actively influence the integrated daycare environment, either by encouraging guests to join in activities and cultivating friendships with them, or—in the more contrary cases—by criticizing negative aspects associated with the presence of daycare guests. Other residents underscored their indifference to the presence of daycare guests. The reluctance of the study participants to address undesirable aspects associated with the daycare guests indicates that some of them are afraid to criticize institutional rules, procedures, and decisions or have not learned to do so.

Our results imply that nursing home staff need to be sensitive to anger and dissatisfaction about the presence of daycare guests, as well as to conflicts between the two user groups. To avoid an entrenchment of negative attitudes, it would be important to react quickly to expressions of annoyance and unhappiness and seek dialogue with all involved. User orientation in the integrated daycare concept can be strengthened by taking the needs, worries, and expectations of residents into account.

### Implications for Future Research

This study reveals the diverse and complex perspectives of nursing home residents on peer relationships as a central element of their living and care situation, from which recommendations can be drawn for refining and improving the integrated daycare model. The study also points to a need for further research on ways to promote inclusion and participation in the nursing home context and ameliorate exclusionary mechanisms. Despite real progress achieved in improving the quality of nursing home services, overcoming the isolation of nursing homes and their residents from the community remains a challenge. The risk of social isolation of nursing home residents has grown with the increase in their burden of disease. Research should investigate in greater depth how residents form and shape their social relationships, and seek models and concepts that can successfully integrate nursing homes into society.

On the other hand, our study underlines the importance of avoiding treating nursing home residents as basically passive recipients of care services. Instead they need to be given a stronger voice as active participants in the nursing home setting—and that means in research contexts too. Unfortunately, there is still a tendency to concentrate on gathering data that reflect the provider perspective and are supposedly “easier” to obtain. But the external and supposedly objective perspective offered by these data cannot substitute the subjective views and experience of residents.

### Limitations of the Study

Most of the participating residents had few cognitive impairments, placing limits on the applicability of our findings. Because residents with severe cognitive impairments had to be excluded from the study, no assertions can be made about their perceptions of the daycare guests’ presence. It is difficult to discern to what extent family members interviewed on behalf of spatially disoriented and/or cognitively impaired residents correctly assessed their relatives’ perceptions. Another consideration is that interviewees may have answered in a manner that they viewed as socially desirable. A supplementary observational study would have been beneficial and should be considered in further research.

## Supplementary Material

gnaa157_suppl_Supplementary_Material
