# Supplementary material for: Between Social Inclusion and Exclusion: Integration of Daycare Guests in the Nursing Home Setting
Source: Gerontologist. 2020 Oct 9;61(7):1030–40. doi: 10.1093/geront/gnaa157 (PMC8437502; doi:10.1093/geront/gnaa157)
Supplement: gnaa157_suppl_Supplementary_Material [file gnaa157_suppl_supplementary_material.docx]

**Online Supplementary Material for Accepted Publication in *The Gerontologist*:**

**Between Social Inclusion and Exclusion: Integration of Daycare Guests in the Nursing Home Setting**

Kerstin Hämel^*^, Dr. rer. soc., Gundula Röhnsch, Dr. phil.

School of Public Health, Bielefeld University, Bielefeld, Germany

*Address correspondence to: Prof. Dr. Kerstin Hämel, School of Public Health, Bielefeld University, Universitätsstraße 25, 33615 Bielefeld, Germany. E-Mail: [kerstin.haemel@uni-bielefeld.de](mailto:kerstin.haemel@uni-bielefeld.de)

**Guideline for Interviewing Home Residents**

**Start of interview**

Some time ago, this facility provided the opportunity for daycare guests to visit this facility during the day. As opposed to yourself and the other residents, day(care) guests do not live in the facility: they have their own flat or house, are cared for/looked after here during the day and leave the facility again in the evening.

During this interview that we are now having I will ask you about how you experience the presence of day(care) guests in this facility. I will keep asking you to relate situations to me, which will illustrate this. There are no “correct” or “wrong” answers in this interview. What is important to me are your own personal perceptions and experiences. All details that would allow any conclusions as to yourself or your family will be pseudonymized, e.g. by stating different names or different times and locations. The scientific evaluation of your statements will be performed under strict compliance with data protection and confidentiality.

If you do not feel able or do not wish to answer any of the interview questions, please let me know accordingly, this is quite normal. Please take your time with your answers. We are in no rush, I am here and I am listening to you.

1. We could start the interview with you telling me about what usually happens in the facility during the day.

- Would you please tell me what you usually do during the day?
- What do you like about it and could you relate a relevant situation to me?
  - Is there anything at all you do not like about it?
- Are there any situations, in which you feel particularly comfortable in the facility? Could you relate one of those situations to me?

1. What do you think, do you have a say in how you spend the day in the facility and in what you do with the other residents and guests? Is there a specific situation to illustrate this?

- Has anything changed recently in your daily routine within the facility? What exactly and could you relate one situation to me?
  - What do you think are the reasons for those changes?

*Filter: Question 3a will only be posed to residents, who have already been living in this nursing home* ***prior to*** *the introduction of ITP.*

3a) If you think back, can you remember the situation when you noticed that something was different in the facility, because all of a sudden there were people there, who arrived in the morning and left in the evening?

- What was the situation then, could you tell me more details?
- Do you remember how you felt in that situation?
- There have been more people here recently than before. How do you feel about this, could you relate a relevant situation to me?
- How do you feel about people being here in during the day and going home again in the evening?

*Filter: Question 3b will only be put to residents, who moved into the nursing home* ***after*** *the introduction of ITP.*

3b) If you think back, can you remember the situation when you noticed that there were day guests in this facility as well, who arrive in the morning and leave again in the evening?

- What did you think then? Would you please tell me more about it?
- How do you feel about people being here during the day and going home again in the evening?

1. Do you feel that due to the presence of people, who come here **for the day** and go home again in the evening, anything has changed for you in this facility?

- Is there anything that you do together? Could you describe in more detail, how this usually occurs?
- How do you like being together with those people who come here for the day but are at home for the night?
- What exactly do you like about it?
- And is there anything that you do not like about this way of living together? Are there any situations that you could relate to me?

1. Quite a different question now: How would you describe the way you are living side-by-side with the other residents, i.e. with those people that are living here constantly?

- Do you think that this co-residence has changed recently? Could you tell me about a situation to illustrate this?
  - What do you think is the reason that the co-residence has changed at all?

1. We have now spoken at length about your co-residence with other people here in this facility. I would now like to address a different subject.

When you think about it: Do you believe that the care you receive has changed in any way recently? What exactly has changed, could you specify this in detail?

- What do you think is the reason that (insert aspect) has improved/deteriorated?

*Filter: The following subquestion is only asked, if the interviewee is of the opinion that* ***nothing*** *regarding his/her care has changed.*

- Have you recently experienced a situation when you did not feel well looked after here? What was the specific situation, could you tell me more about it?

1. How would you describe your relationship with the nursing staff in this facility?

- In general: What would a ‘good’ nurse be like in your eyes? Could you name an example to illustrate this?
- Is there such a member of nursing staff in this facility?
- Are you of the opinion that anything has recently changed in your relationship with that person? Could you describe this to me with an example?
  - What is the reason for this change of relationship in your view?
  - Has your relationship with the other nursing staff members changed as well?

1. Have you ever complained to any of the nursing staff about anything in this facility?

- What was it exactly, do you remember?
- And how did the nursing staff members react to the complaint?
- Do you think that anything has changed regarding (insert incident that the complaint was based on)?

1. We have almost come to the end of this interview now. Thank you very much for taking the time and telling me so much.

- I have one last question to ask: Apart from the subjects we discussed today is there anything else that we have not spoken about yet, but that you would like to address yourself?

**Guideline for Interviewing Relatives of Home Residents**

**Start of interview**

Some time ago, this facility provided the opportunity for daycare guests to visit this facility during the day. As opposed to your relative/s and the other residents, day(care) guests do not live in the facility: they have their own flat or house, are cared for/looked after here during the day and leave the facility again in the evening.

During this interview that we are now having I will ask you about your opinion as to how your relative experiences the presence of daycare guests in this facility. I will keep asking you to relate situations to me, which will illustrate this. There are no “correct” or “wrong” answers in this interview, what is important to me are your own personal perceptions and experiences. All details that would allow any conclusions as to yourself or your family will be pseudonymized, e.g. by stating different names or different times and locations. The scientific evaluation of your statements will be performed under strict compliance with data protection and confidentiality. If you do not wish or do not feel able to answer any of the interview questions, please let me know accordingly, this is not a problem at all.

1. We could start the interview with you telling me about what your relative usually does in the facility during the day.

- Do you think that your relative likes it? How does this become evident?
- And vice versa: Is there anything that your relative does not like too much?
- Are there any situations, in which your relative appears to feel particularly comfortable in the facility? Is there a specific situation to illustrate this?

1. What do you think, does your relative have a say in how he/she spends the day in the facility and what he/she does with the other residents and guests?

- In your opinion, has anything changed recently in your relative’s daily routine within the facility? What exactly and could you relate one situation to me?
  - What do you think are the reasons for those changes?

*Filter:* ***Question 3a*** *will* ***only*** *be posed to* ***relatives****, whose relative has already been living in this nursing home* ***prior to*** *the introduction of ITP.*

3a) Do you think that your ….. noticed that something was different in the facility, because all of a sudden there were people there, who arrived in the morning and left in the evening?

- - Could you tell me, how your ….. perceived those changes in the facility?
- In your opinion, how does your ….. feel about the fact that there are people, who are here during the day but go home again in the evening?
- Owing to the day guests there have been more people here recently. How do you think your …. feels about this, could you relate a situation to me?

*Filter: Question 3b will only be put to relatives, whose relative moved into the nursing home after the introduction of ITP.*

1. If you think back, can you remember the situation, when your relative noticed that there are also day guests in this facility, who arrive in the morning and leave again in the evening?

- How did your relative address this topic, could you relate this situation to me?
- How, in your opinion, does your relative feel about the fact that there are people who are here **during the day** but go home again **in the evening**?

1. Do you think that due to the presence of people, who come here for the day and go home again in the evening, your relative feels that this facility is special?
2. In your opinion, how does your relative like to spend time with people, who are only here during the day but are at home over night?
   - What exactly does he/she like about it?

- And is there anything that your relative does not like about this form of being together? Is there a relevant situation that you could relate to me?

1. Quite a different question now: How would you describe the way your relative is living side-by-side with the other residents anyway?

- Do you think that anything has changed recently in this co-residence? Could you tell me about a situation to illustrate this?
  - What do you think is the reason that the co-residence has changed at all?

1. I would now like to address a different subject. When you think back: Do you believe that the care your relative receives has changed in any way? What exactly has changed, could you specify this in detail.

- What do you think is the reason that (insert here aspect stated by the interviewed person) has improved/deteriorated?

*Filter: The following subquestion is only asked, if the interviewee is of the opinion that* ***nothing*** *regarding the care has changed for his/her relative.*

- Has your relative recently mentioned that he/she did not feel well looked after here? What was the specific situation, could you tell me more about it?

1. How would you describe the relationship between your relative and the nursing staff in this facility?

- In your opinion, what does your relative expect from the nursing staff in this facility? What would a ‘good nurse’ be like in his/her eyes?
- Do you think that there is indeed such a member of nursing staff here?
- Are you of the opinion that anything has recently changed in the relationship between your relative and that person? What would you link this to?
  - What is the reason for this change of relationship in your view?
- Do you believe that the relationship between your relative and the other nursing staff members changed as well?

1. Has your relative or have you ever complained to any of the nursing staff about anything in this facility?

- What was it exactly, could you relate this situation to me?
- And how did the nursing staff members react to the complaint?
- Do you think that anything has changed regarding (insert incident that the complaint was based on)?

1. We have almost come to the end of this interview now. Thank you very much for taking the time and telling me so much.

- I have one last question to ask: Apart from the subjects we discussed today is there anything else that we have not spoken about yet, but that you would like to address yourself?
